# Label‐Free 3D Photoacoustic Imaging of Tumor Organoids for Volumetric Drug Screening

**DOI:** 10.1002/advs.202417226

**Published:** 2025-06-04

**Authors:** Xiaofei Luo, Maike Chen, Han Shan, Xize Yu, Qibo Lin, Qian Tao, Xiongwei Wei, Changling Lv, Ziyan Chen, Fan Zhuo, Xi Zhu, Jiaxing He, Zhaoxi Li, Chunlong Fei, Jing Xu, Juan Su, Zeyu Chen, Shuang Zhao, Xiang Chen

**Affiliations:** ^1^ Department of Dermatology Xiangya Hospital Central South University Changsha 410008 China; ^2^ Department of Mechanical and Vehicle Engineering Changsha University of Science and Technology Changsha 410114 China; ^3^ National Engineering Research Center of Personalized Diagnostic and Therapeutic Technology Changsha 410013 China; ^4^ Furong Laboratory Changsha 410008 China; ^5^ State Key Laboratory of Precision Manufacturing for Extreme Service Performance College of Mechanical and Electrical Engineering Central South University Changsha 410083 China; ^6^ School of Microelectronics Xidian University Xi'an 710071 China; ^7^ Department of Integrated Traditional Chinese & Western Medicine The Second Xiangya Hospital Changsha 410008 China; ^8^ Yunan Key Laboratory of Breast Cancer Precision Medicine Institute of Biomedical Engineering Kunming Medical University Kunming 650500 China; ^9^ Department of Otolaryngology Xiangya Hospital Central South University Changsha 410008 China; ^10^ National Clinical Research Center for Geriatric Disorders Xiangya Hospital Central South University Changsha 410008 China

**Keywords:** label free, photoacoustic imaging, precision medicine, tumor organoids, volumetric drug screening

## Abstract

As one of the most advanced in vitro drug screening platforms, tumor organoids require accurate pharmacosensitivity assessments to ensure reliable results. However, achieving accurate volume assessment of these 3D organoid models remains a challenge in traditional drug screening processes. Here, a label‐free organoids photoacoustic imaging (LFOPI) system is introduced for high‐resolution 3D imaging of tumor organoids. The capabilities of the LFOPI system are evaluated by monitoring structural transformations in melanoma organoids. The LFOPI system is further employed for volumetric drug screening of melanoma tumor organoids. Drug screening with cisplatin and temozolomide reveal that LFOPI‐based organoid volume data correlates more strongly with viability trends (0.8627 and 0.9069) than traditional diameter‐based methods (0.8190 and 0.7849). Additionally, immunotherapy drug screening demonstrates the capability of the system to precisely assess the 3D volumes of irregularly shaped organoids. This LFOPI system provides a promising method for label‐free drug screening and personalized treatment.

## Graphical abstract

A label‐free organoid photoacoustic imaging (LFOPI) system is developed for precise volumetric drug screening evaluation. As a proof of concept, LFOPI's performance in chemotherapy and immunotherapy drug screening assays are further evaluated. The LFOPI offers unique non‐invasive, label‐free capabilities for tumor organoids, and contributes to the development of therapeutic evaluations in cancer research.

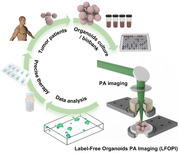

## 1 Introduction

Tumor organoids, derived from tumoral tissues, have profoundly reshaped the landscape of cancer research by offering a more accurate and functional representation of tumors in vitro.^[^ ^1^, ^2^, ^3^ ^]^ These 3D constructs closely mimic the complex architecture and microenvironment of actual tumors, thereby providing a superior platform for oncological studies and therapeutic development compared to traditional 2D cell cultures. Crucially, tumor organoids facilitate the explorations of personalized treatment regimes, a cornerstone in the evolution of precision medicine.^[^ ^4^ ^]^

In the domain of oncology, the use of tumor organoids for drug screening is pivotal,^[^ ^5^, ^6^, ^7^ ^]^ propelling advancements in individualized therapy protocols that target specific patient‐derived tumors. This application is particularly vital as it aligns with the broader goal of enhancing patient outcomes through tailored treatment strategies. Despite their potential, the predominant reliance on viability and 2D growth metrics to evaluate drug responses in tumor organoids constitutes a critical limitation.^[^ ^8^, ^9^, ^10^ ^]^ Such assessments fail to capture the full spectrum of drug effects within the 3D context of tumor organoids, potentially skewing efficacy assessments and therapeutic decisions.

The traditional methodologies for obtaining 3D data from tumor organoids often involve intricate and costly procedures,^[^ ^11^ ^]^ such as confocal microscopy,^[^ ^7^ ^]^ wide‐field structured‐illumination microscopy (SIM),^[^ ^12^ ^]^ light‐sheet microscopy.^[^ ^13^ ^]^ These fluorescence microscopy techniques require dye staining of the samples to provide image contrast. This labor‐intensive approach reconstructs 3D images from serial 2D scans, which is not only time‐consuming but also disrupts the physiological state of organoids due to the requirement for fluorescent labeling. Such invasive methods can impair the viability and functional integrity of the organoids, thereby limiting their further use in dynamic and repeated assessments that are crucial for robust drug testing. To address these challenges, the incorporation of photoacoustic imaging into tumor organoid studies offers a promising technological approach, enabling high‐resolution, label‐free, and volumetric imaging.^[^ ^14^, ^15^ ^]^ Photoacoustic imaging converts absorbed light into ultrasonic waves via thermoelastic expansion, enabling imaging and analysis that reveals the spatial distribution of light absorption based on the detected acoustic signals. In particular, optic‐resolution photoacoustic microscopy (OR‐PAM) is a widely used imaging technique in clinical applications, attributed to the capability of providing high‐resolution and high‐sensitivity visualization of wavelength‐dependent optical absorption at the cellular level.^[^ ^16^, ^17^ ^]^ Depending on the illumination wavelength, label‐free PAM can image various contrasts, including hemoglobin,^[^ ^18^, ^19^ ^]^ melanin,^[^ ^20^ ^]^ DNA/RNA,^[^ ^21^ ^]^ cytochrome,^[^ ^22^ ^]^ lipids,^[^ ^23^ ^]^ and proteins.^[^ ^24^ ^]^ Notably, photoacoustic imaging has also been demonstrated to visualize neuromelanin distribution in brain organoids, further highlighting its potential in organoid‐based biomedical research.^[^ ^25^, ^26^ ^]^ Therefore, this technique allows for direct, non‐invasive, and label‐free 3D imaging, offering a comprehensive view of organoid architecture and function without compromising viability.

In this study, we introduce a label‐free organoids photoacoustic imaging (LFOPI) system designed for rapid 3D imaging of tumor organoids. We conducted volumetric drug screening experiments using melanoma organoids to assess the utility of our LFOPI system in monitoring 3D volume changes during drug exposure. By comparing the results with traditional methods that primarily rely on viability assays and 2D diameter measurements, we observed that LFOPI‐based organoid volume data correlates more strongly with viability trends (0.8627 and 0.9069) than traditional diameter‐based methods (0.8190 and 0.7849). In addition, immunotherapy drug screening suggested the capability of our system to precisely assess the 3D volumes of irregularly shaped organoids. This discrepancy highlights the substantial value of incorporating 3D information into the assessment framework for drug efficacy on tumor organoids. The implementation of our system in volumetric drug screening not only offers more accurate volumetric outcomes but also holds the potential to refine and advance current drug screening methodologies, ensuring a more targeted and precise approach in drug efficacy evaluation.

## 2 Results

### 2.1 LFOPI‐Based Volumetric Drug Screening

**Figure 1:**
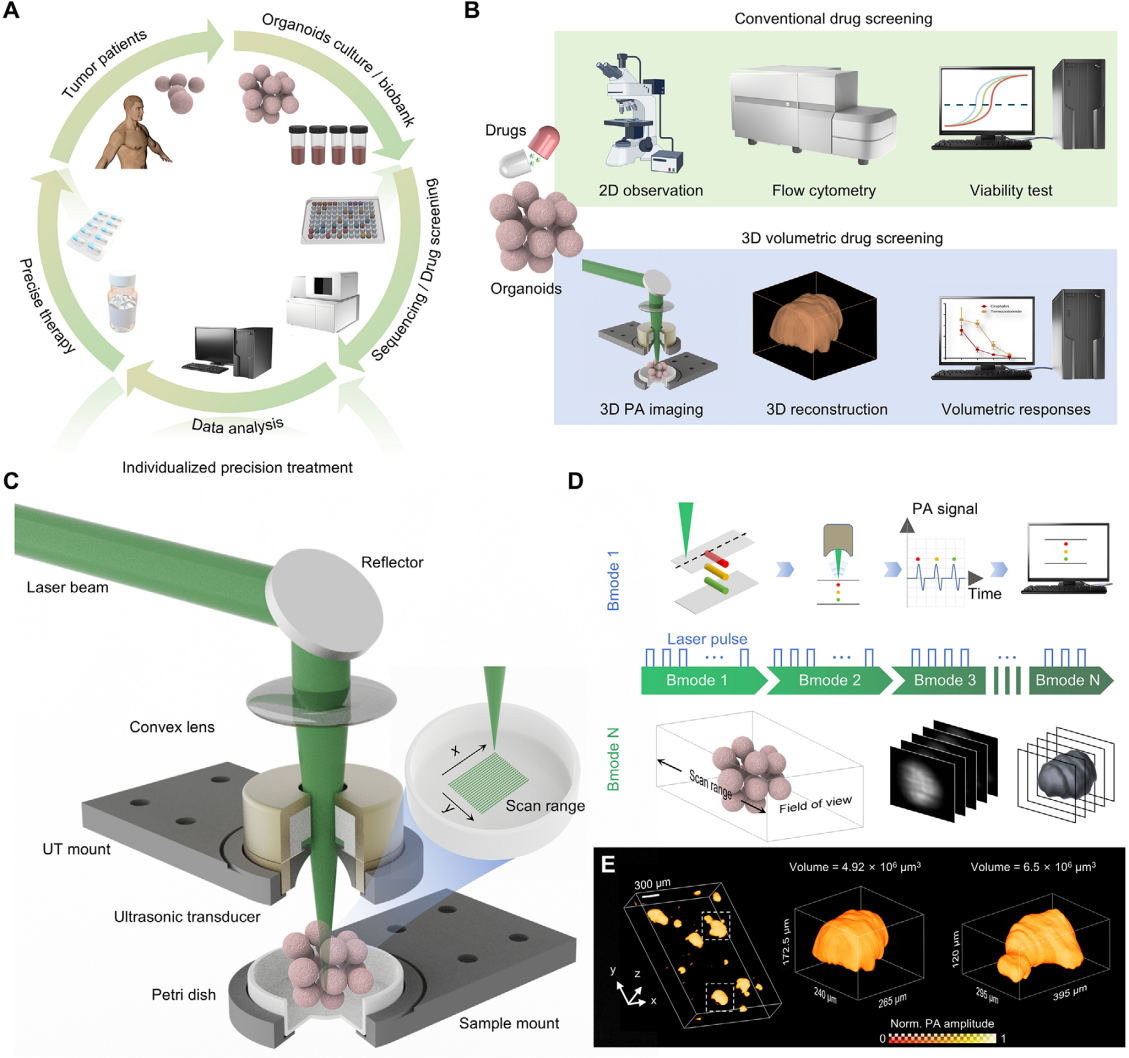
LFOPI system for non‐invasive assessment of volume changes in tumor organoids. A) The workflow of individualized precision treatment for tumor patients. B) The comparison of conventional drug screening and our LFOPI‐based volumetric drug screening. C) Schematic of the LFOPI system for 3D photoacoustic imaging of tumor organoids. D) Principles of volume calculation in the LFOPI system. A laser pulse generates ultrasound signals that form an A‐line. Linear scanning of the sample produces a B‐scan, while planar scanning results in a 3D C‐scan. Subsequent thresholding segments the sample, and volume is calculated based on pixel counts. E) Representative 3D photoacoustic images of melanoma organoids.

As illustrated in Figure 1A, drug screening based on tumor organoids is a pivotal component in the personalized diagnosis and treatment of cancer patients. Current protocols for drug screening using 3D organoids, as depicted in Figure 1B, typically involve viability assessments and the measurement of dimensions on a 2D plane via microscopy. However, due to the irregular external dimensions of 3D tumor organoids, tumor volumes estimated from 2D observations are often inaccurate. Notably, our LFOPI system overcomes these limitations through its label‐free, non‐invasive 3D imaging capabilities. By providing accurate spatial information, it enables reliable 3D quantification of tumor volume, which shows markedly improved precision over conventional 2D estimations. Based on this advantage, volumetric drug screening has emerged as a promising strategy for evaluating drug sensitivity through dynamic measurement of volumetric changes in 3D organoids. Our LFOPI system provides an efficient technique for capturing the 3D structure of tumor organoids, as shown in Figure 1C. Photoacoustic imaging, the principle underlying the LFOPI system, involves the generation of acoustic waves through the absorption of optical energy by the target, followed by thermal expansion. This technique is especially suited to the non‐invasive imaging of biological tissues, as it allows for deep tissue penetration and high‐contrast imaging based on the optical properties of the tissue components. The LFOPI system utilizes a 532 nm nanosecond pulsed laser, which is well‐absorbed by melanin, enhancing contrast and enabling precise delineation of tumor organoid boundaries and structures. This choice of wavelength not only improves contrast but also allows for higher spatial resolution compared to longer wavelengths such as 800 or 1064 nm,^[^ ^15^ ^]^ which is crucial for the detailed visualization of organoid features and intricate details. Compared to shorter wavelengths (e.g., below 500 nm), 532 nm offers advantages such as reduced optical scattering and improved penetration through the 3D culture matrix, while still maintaining high spatial resolution. These characteristics make it easier to obtain stable and reproducible signals in our imaging context. The LFOPI system is particularly effective for direct 3D imaging of melanoma organoids surfaces, facilitating rapid pathological diagnosis and drug evaluation. Given that the height of organoid samples is approximately 100 µm—smaller than the depth of focus (DOF) of our system, which is about 120 µm—the real‐time 3D scanning capability of the LFOPI system ensures that the imaging location remains within the DOF, thereby maintaining a consistent, diffraction‐limited lateral resolution for surface imaging of organoids. The achieved lateral resolution is 7.5 µm.

After acquiring a 3D LFOPI image, we apply principles of linear algebra and geometric volume calculations, aggregating discrete voxels to estimate the overall sample volume. This volumetric analysis enables a detailed representation of the drugs impact on organoid volume (Figure 1D). Figure 1E presents 3D Photoacoustic images of tumor organoids obtained using the LFOPI system, further demonstrating that LFOPI is an ideal method for acquiring comprehensive 3D information from organoid models. This method provides a more accurate reflection of drug efficacy, enhancing the predictive power of preclinical models in oncology.

### 2.2 Direct 3D Reconstruction of Melanoma Organoids via LFOPI

**Figure 2:**
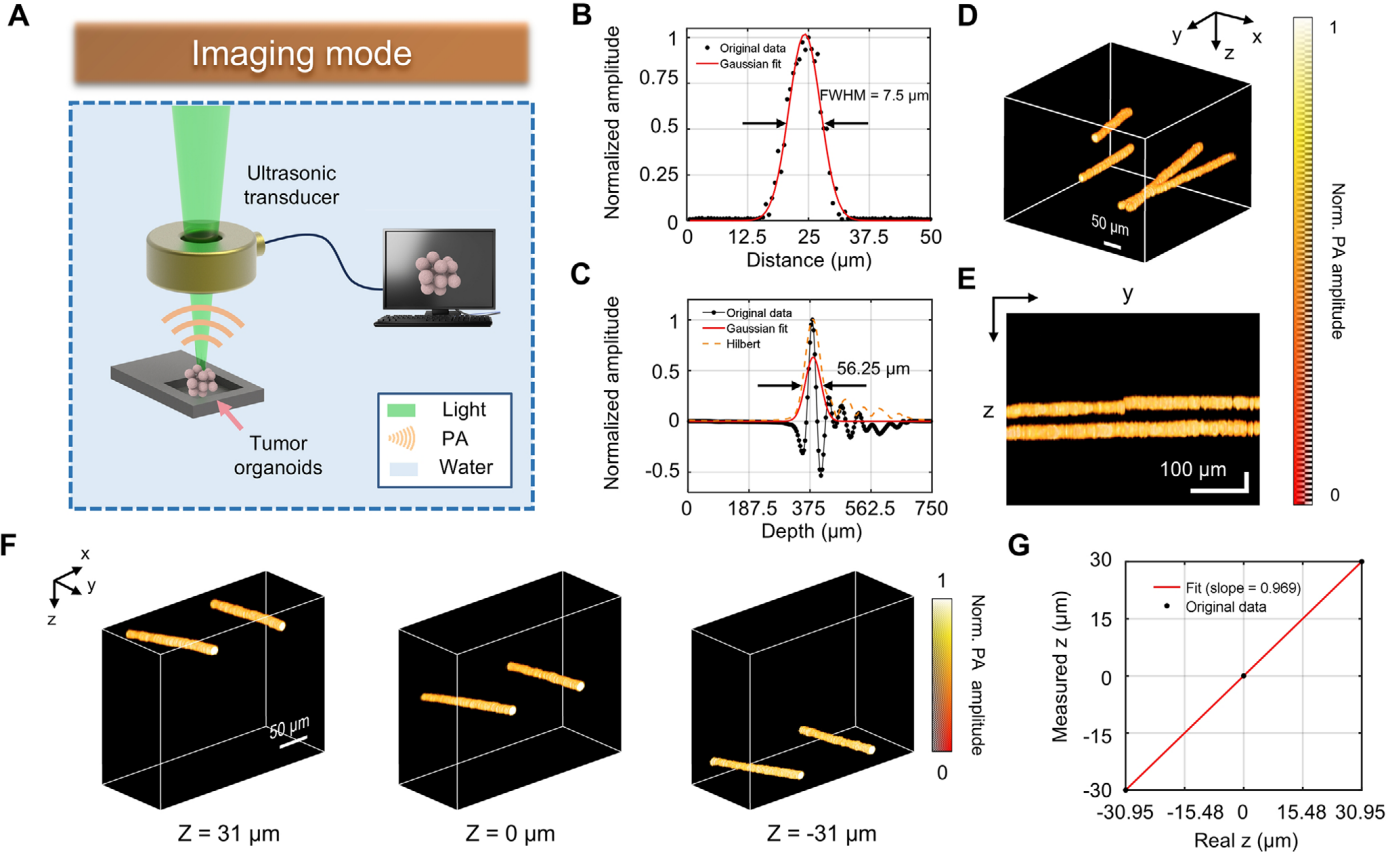
Spatiotemporal characterization of the LFOPI system. A) Illustration of the imaging mode in LFOPI. B) Calculation of lateral resolution of the system. C) Calculation of longitudinal resolution of the system. D) The 3D photoacoustic image of four fiber in agar. E) Maximum x‐projections of the 3D volume in D. F) A set of 3D reconstructions of two fibers at different z‐positions. G) Linear correlation plot comparing the measured z positions from LFOPI to the actual z positions.

Figure 2A,B illustrate the schematic setup and imaging mode of the LFOPI system, respectively. Figure S1 (Supporting Information) shows the system configuration, where a laser source generates nanosecond pulses that are directed towards the target via an arrangement of lenses and a pinhole. In order to achieve precise observations of organoids, our LFOPI system incorporates a hollow focused ultrasonic transducer (Figure S2, Supporting Information), designed specifically for high‐sensitivity reflective optical resolution photoacoustic imaging. The three‐axis motorized stage holds the sample and allows for precise manipulation in the x, y, and z directions, enabling comprehensive scanning and imaging. The imaging mode of the system is depicted in Figure 2A. The sample is submerged in a water tank to facilitate the transmission of photoacoustic waves generated by the laser‐induced thermoelastic expansion of the melanin within the organoids. An ultrasonic transducer positioned above the sample captures these photoacoustic signals, which are then amplified and sent to a data acquisition unit (DAQ). The DAQ digitizes the acoustic signals which are then processed by the connected computer to reconstruct a 3D image of the organoid.

Spatiotemporal is a critical parameter of the LFOPI system, determining the ability to achieve high‐precision 3D photoacoustic imaging of tumor organoids. To quantify the spatiotemporal of the LFOPI system, we imaged a carbon fiber embedded in an agar block. Figure 2C shows the lateral profile of photoacoustic signals with scanning step size of 0.625 µm. The profile is fitted with a line spread function, from which the full width at half maximum (FWHM) is extracted as the lateral resolution, measured at 7.5 µm. Figure 2D displays the photoacoustic A‐line signal from the carbon fiber following total impulse response (TIR) correction and its corresponding Hilbert transform (envelope detection).^[^ ^27^ ^]^ The axial resolution, determined from the FWHM of the envelope, is assessed to be 56.26 µm. To explore the 3D imaging capabilities of the LFOPI system, we imaged four carbon fibers embedded within an agar block (shown in Figure 2D). The fibers were strategically positioned in close proximity to each other, creating a simple geometric pattern observable in x‐projections (Figure 2E). To further validate the accuracy of the 3D volumes reconstructed by the LFOPI system, we imaged two carbon fibers submerged in water. The z‐positions of the carbon fibers were precisely adjusted and measured using a linear translation stage. By imaging the carbon fibers at multiple z‐positions and reconstructing the 3D volumes (as depicted in Figure 2F), we compared the z‐positions in the reconstructed volumes with their actual measurements. Utilizing optical focusing and time‐of‐flight information derived from the photoacoustic signals, LFOPI effectively captured the contrast distribution along the axial (z) direction. The correlation between the measured and actual z‐positions, as shown in Figure 2G, was linear (*R* ^2^ = 1) with a slope close to 1, confirming the high fidelity in reconstructing 3D objects axially. The results demonstrate that the LFOPI system not only accurately reconstructs the 3D structures but also provides a robust means of examining intricate spatial relationships within the sample. The high precision of our system is crucial for applications requiring detailed structural analysis, such as in biomedical imaging and 3D reconstruction, where understanding the microarchitecture of complex structures is essential.

**Figure 3:**
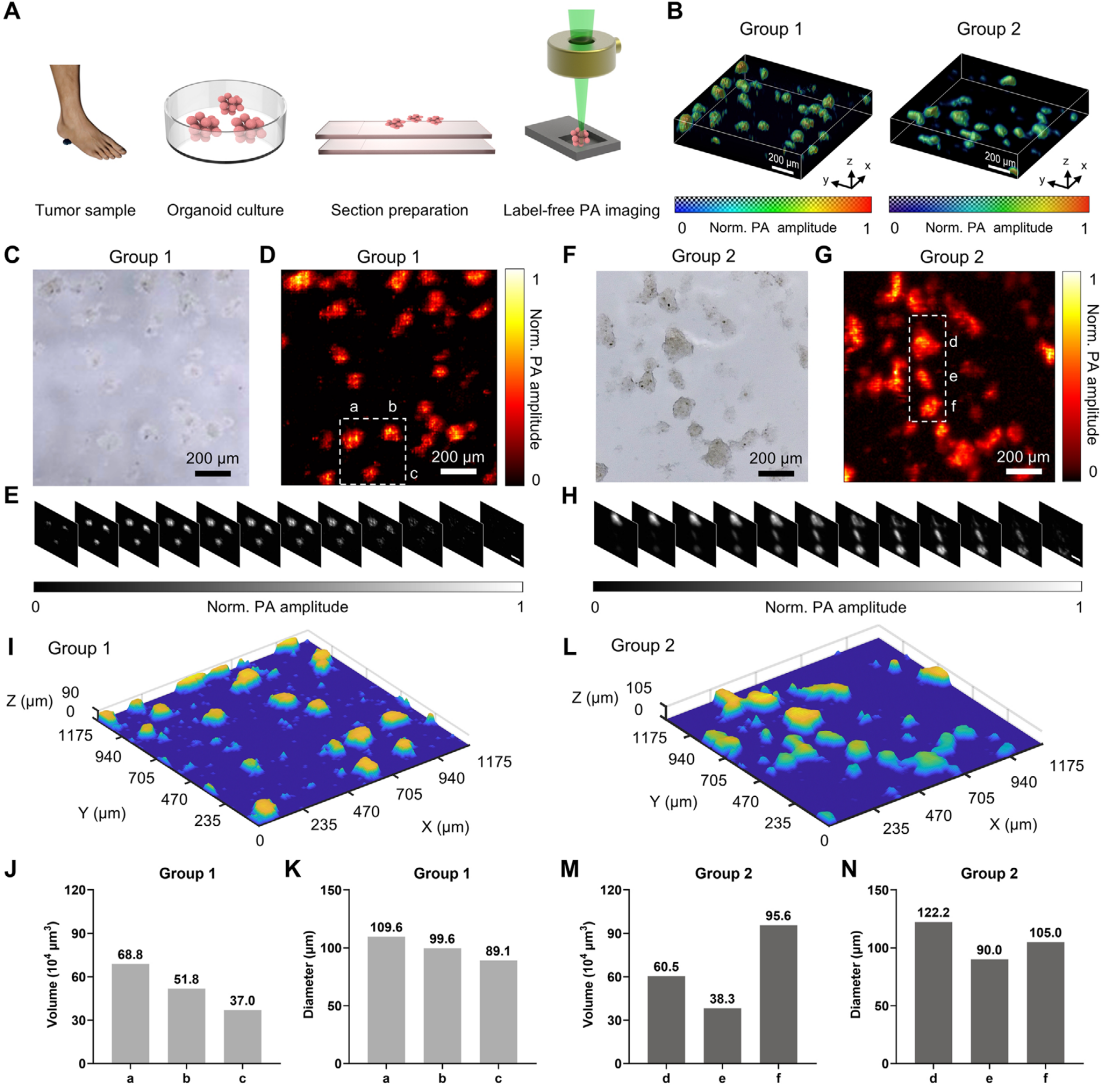
3D label‐free photoacoustic imaging of melanoma organoids. A) Workflow of the 3D photoacoustic imaging process for melanoma organoids. B) 3D reconstructions of melanoma organoids by the LFOPI system. C,F) Bright field microscopy images of melanoma organoids before photoacoustic imaging. D) and G 3D photoacoustic images of two melanoma organoid samples. E,H) Series of z‐stack images showing the depth‐resolved imaging capability of the LFOPI system for group 1 and group 2, with normalization of photoacoustic amplitude from 0 to 1. Scale bars, 100 µm. I,L) The melanoma organoids surface profiles of group 1 and group 2, respectively. J,M) Volume histograms for samples a‐c and d‐f, respectively. K,N) Diameter histograms for samples a–c and d–f, respectively.

To elucidate the capabilities of the LFOPI system, we further established patient‐derived melanoma organoids. For testing convenience, the melanoma organoids were positioned on a custom‐designed, water‐immersed sample holder for imaging (Figure 3A). We conducted 3D imaging on two distinct groups of organoid samples (Figure 3B,D,G). To validate the 3D photoacoustic imaging results, we also used an optical microscope to observe the morphology of the organoid (Figure 3C,F). The results indicated that the morphology of the organoids observed under the optical microscope was highly consistent with the photoacoustic images, suggesting that our system can accurately reconstruct the 3D structure of the organoids. The corresponding photoacoustic images obtained through raster‐scanning (Figure 3D,G) demonstrated consistent resolution across the field of view (FOV). This consistency is crucial as it allows for accurate comparisons across different samples and imaging sessions, enhancing the reliability of the LFOPI as a diagnostic tool. To quantitatively assess the volume of tumor organoids, we selected three samples from each of two groups for quantitative calculation. Detailed views of melanoma organoids at different z positions are shown in Figure 3E (sample a, b, and c) and Figure 3H (sample d, e, and f), derived from the respective larger scans in Figure 3D,G. Surface profiles of the melanoma organoids were reconstructed using the time‐of‐flight information from the photoacoustic signals (Figure 3I,L), which unveiled minor fluctuations in surface height and confirmed that over 90% of the pixels fell within the DOF. To quantitatively assess the volume of the melanoma organoids, we calculated the volume of organoids by aggregating discrete voxels, thus representing the overall sample volume (Figure 3J,M), and determined the sample diameter (Figure 3K,N). This quantitative measurement is instrumental for correlating organoid size with drug sensitivity and monitoring changes over time. Given the non‐destructive nature of the LFOPI method, these intact melanoma organoids were subsequently available for further drug screening and pathological examination. This approach facilitates the direct assessment of tumor characteristics and therapeutic responses by monitoring the volume changes of organoids.

### 2.3 Volumetric Drug Screening for Evaluating the Chemotherapy and Immunotherapy Efficiencies

**Figure 4:**
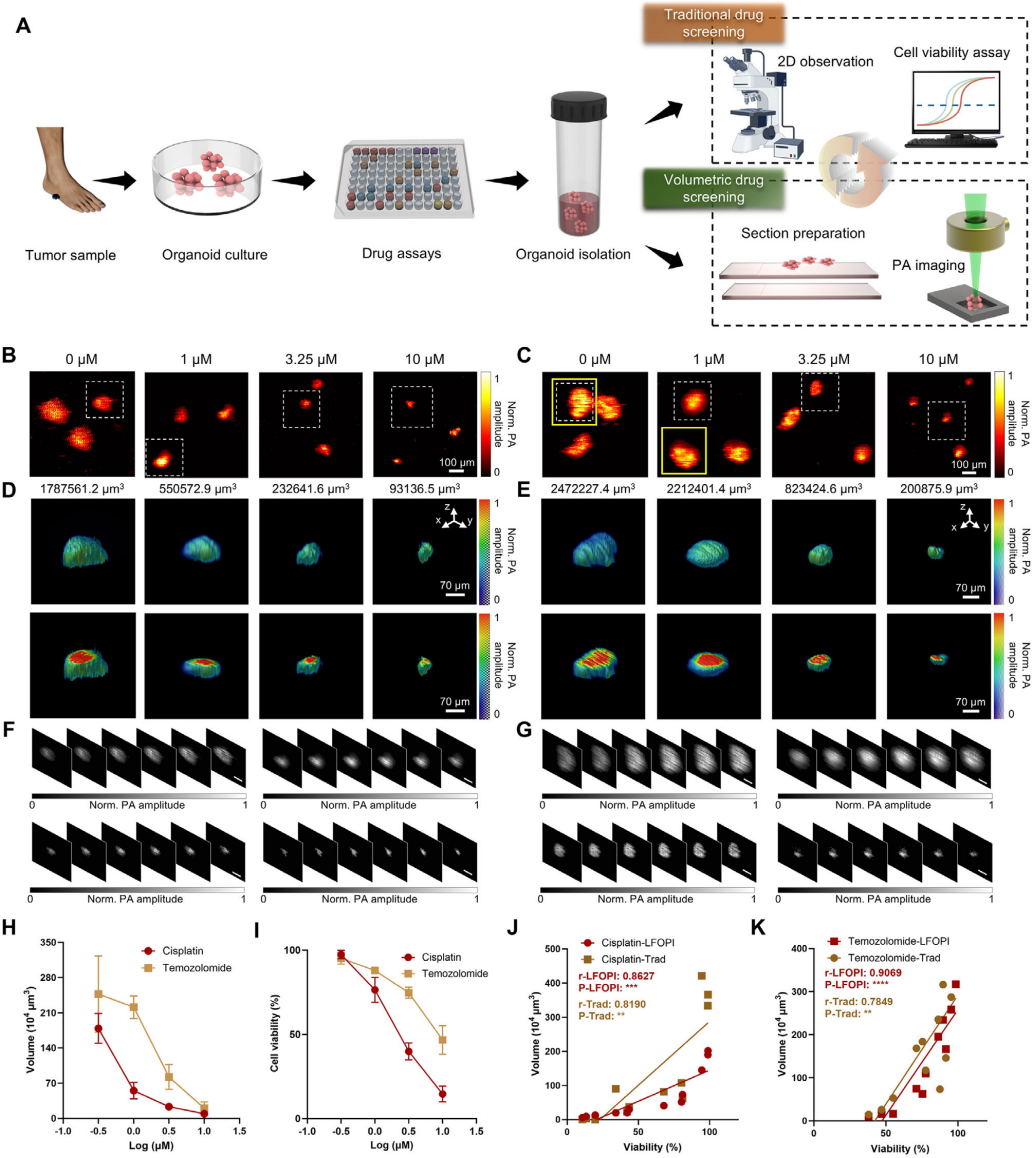
Volumetric chemotherapy drug screening with LFOPI. A) Workflow illustrating the process of 3D tumor organoid chemotherapy drug screening using LFOPI. B) Maximum z‐projections of the 3D volumes of organoids treated with cisplatin across four treatment groups: 0 µm (control), 1 µm, 3.25 µm, and 10 µm drug concentration groups. C) Maximum z‐projections of the 3D volumes of organoids treated with temozolomide across the same four treatment groups. The tumor organoids highlighted by the yellow boxes in the control and 1 µm groups exhibit similar 2D projected areas (35775 µm^2^ and 36825 µm^2^, respectively), yet their volumes differ markedly (3171375 µm^3^ and 2341875 µm^3^, respectively). D,E) Detailed 3D volume and cross‐sectional views (indicated by white dots) of the organoid groups treated with cisplatin and temozolomide, respectively. F,G) Sequential z‐position slices from the 3D reconstructions shown in (D,E), demonstrating depth‐resolved imaging capabilities. Scale bars, 100 µm. H) Dynamic volumetric responses across drug dosages. Data are presented as mean ± s.d. (*n* = 3). I) Cell viability of tumor organoids treatment with varying concentrations of cisplatin and temozolomide. Data are presented as mean ± s.d. (*n* = 3). J,K) Correlation between organoid volumes estimated by LFOPI and traditional methods with cell viability after treatment with cisplatin and temozolomide.

Enabled by the non‐invasive, label‐free 3D imaging capabilities of the LFOPI system, we anticipated that this technology would be ideally suited for monitoring the efficacy of chemotherapy drugs. Our motivation for conducting this study stemmed from the need to better understand the dynamic responses of melanoma organoids to chemotherapeutic agents, which could ultimately guide more effective treatment strategies. Figure 4A illustrates the process and outcomes of our use of the LFOPI system for volumetric melanoma organoids chemotherapy drug screening. We cultured patient‐derived melanoma organoids and divided them into four cohorts: a control group (untreated) and three experimental groups treated with ascending concentrations of cisplatin and temozolomide. We chose these drugs because cisplatin is known for its efficacy in causing DNA crosslinking and apoptosis, while temozolomide acts as an alkylating agent that requires cellular replication for its cytotoxic effects. Understanding their distinct mechanisms of action on melanoma organoids could help in tailoring chemotherapy treatments. In addition to their different modes of action, we selected cisplatin and temozolomide based on their well‐recognized clinical relevance. Cisplatin continues to be a cornerstone in the treatment of advanced melanoma, often used in combination therapies, whereas temozolomide is the only FDA‐approved oral alkylating agent and represents a standard‐of‐care option. Their well‐established roles in current treatment paradigms provide a robust framework for assessing differential drug sensitivities in patient‐derived organoids.

In our workflow, we captured the dynamic response of these organoids to the treatments. Figure S3 (Supporting Information) show bright field microscopy images of melanoma organoids treated with ascending concentrations of cisplatin and temozolomide. The maximum z‐projections of the 3D tumor volumes we obtained showed clear structural differences between untreated organoids and those treated with cisplatin (Figure 4B) and temozolomide (Figure 4C). Untreated organoids retained their original size and morphology, whereas the treated groups exhibited marked morphological changes with increasing drug concentrations, characterized by reduced tumor volume at higher dosages. To further illustrate this point, Figure 4C highlights two tumor organoids within the yellow boxes from the 0 µm temozolomide control group and the 1 µm temozolomide treatment group, which exhibit nearly identical projected areas—35775 and 36825 µm^2^, respectively. If estimated from 2D area alone, their volumes would appear similar. However, LFOPI reveals a marked volumetric difference between the two: 3171375 µm^3^ for the control group and 2341875 µm^3^ for the treatment group. This example underscores the limitation of 2D projections in volumetric assessment and demonstrates LFOPI's capability to resolve subtle yet critical changes along the z‐axis.

Figure 4D,E provides detailed views of specific regions within the organoids, accentuating the finer‐scale impacts of chemotherapy, particularly in areas displaying cell death and disintegration. This precise visualization of cell structure and tumor architecture is crucial for understanding how organoids respond to chemotherapy at a cellular level. Figure 4F,G presents slice images from these figures at different z‐positions, allowing us to precisely localize tumor regions affected by the drugs. We followed this 3D photoacoustic imaging with quantitative analyses, incorporating cell viability assays that showed a clear decrease in viability as drug concentrations increased, which strongly correlated with the changes observed in the LFOPI images. To independently validate our findings beyond traditional viability assays, we performed live/dead staining using AO/PI dyes to assess cell viability. The collected data and analysis show a trend consistent with traditional viability assays (Figure S6, Supporting Information). Our measurements of tumor diameter and volume indicated a notable trend of shrinkage in the treated groups, particularly evident in those exposed to high concentrations of cisplatin, reflecting the potent efficacy of drugs. Figure 4H,I visually and quantitatively depicts how tumor volumes and cell viability responded to the differing concentrations of cisplatin and temozolomide.

Figure 4H illustrates the differential responses in tumor organoid volume under varying concentrations of cisplatin and temozolomide. Specifically, at low drug concentrations, the organoid volume in the cisplatin‐treated group was reduced to just 24.9% of the volume of those treated with temozolomide. At medium drug concentrations, the tumor volume in the cisplatin‐treated group shrank to only 28.3% of those in the temozolomide‐treated group. At high concentrations, the tumor volumes further diminished, with those treated with cisplatin reducing to 46.4% of the volumes observed in the temozolomide group, clearly demonstrating the more potent anti‐tumor efficacy of cisplatin. Figure S4 (Supporting Information) shows the comparison of organoid volumes obtain by traditional diameter‐based method and LFOPI method. We found that LFOPI‐based volume values were smaller than those of traditional method. This observation underscores the importance of using our LFOPI system for volumetric drug screening, as it provides more accurate 3D volume data of tumor organoids. This differential reduction in tumor volume can be attributed to the distinct mechanisms of action of the two drugs. Cisplatin, a platinum‐based chemotherapeutic agent, exerts its effects by forming DNA adducts and cross‐links that prevent DNA replication and transcription, triggering apoptosis. This mechanism is particularly effective against rapidly dividing tumor cells, accounting for the substantial volume reduction observed even at lower concentrations. In contrast, the more gradual effects of temozolomide illustrated its dependency on the cell cycle for efficacy. This cell‐cycle dependency is likely why we observed a slower and less pronounced reduction in tumor volume at lower concentrations of temozolomide. Figure 4I presents the cell viability data corresponding to various concentrations of cisplatin and temozolomide, mirroring the trends observed in tumor volume changes. However, we observed that changes in tumor volume manifested more rapidly than changes in cell viability. This suggests that the LFOPI system can detect alterations in the 3D volume of tumors before noticeable effects on cell viability are apparent, highlighting its sensitivity and capability for early detection in assessing the impact of chemotherapy drugs. The IC50 values indicated (the concentration of drug at which cell viability is reduced to 50%) suggest that cisplatin has a stronger cytotoxic effect compared to temozolomide across the concentrations tested (Figure S4, Supporting Information). Figure 4J,K presents Pearson correlation analyses between volume and viability following treatment with cisplatin and temozolomide, respectively. The correlation coefficients (*r*‐values) indicate a strong positive correlation between organoid volume and viability, particularly for organoid volumes obtained by the LFOPI (0.8627 vs 0.8190 and 0.9069 vs 0.7849, respectively). This suggests that the LFOPI enhances the precision of organoid volume measurements, thereby providing more reliable data for evaluating the efficacy of anticancer drugs. This method offers substantial advantages for volumetric drug screening, allowing us to visualize and quantify drug effects in real‐time, which is critical for advancing personalized treatment strategies and improving outcomes for cancer patients.

**Figure 5:**
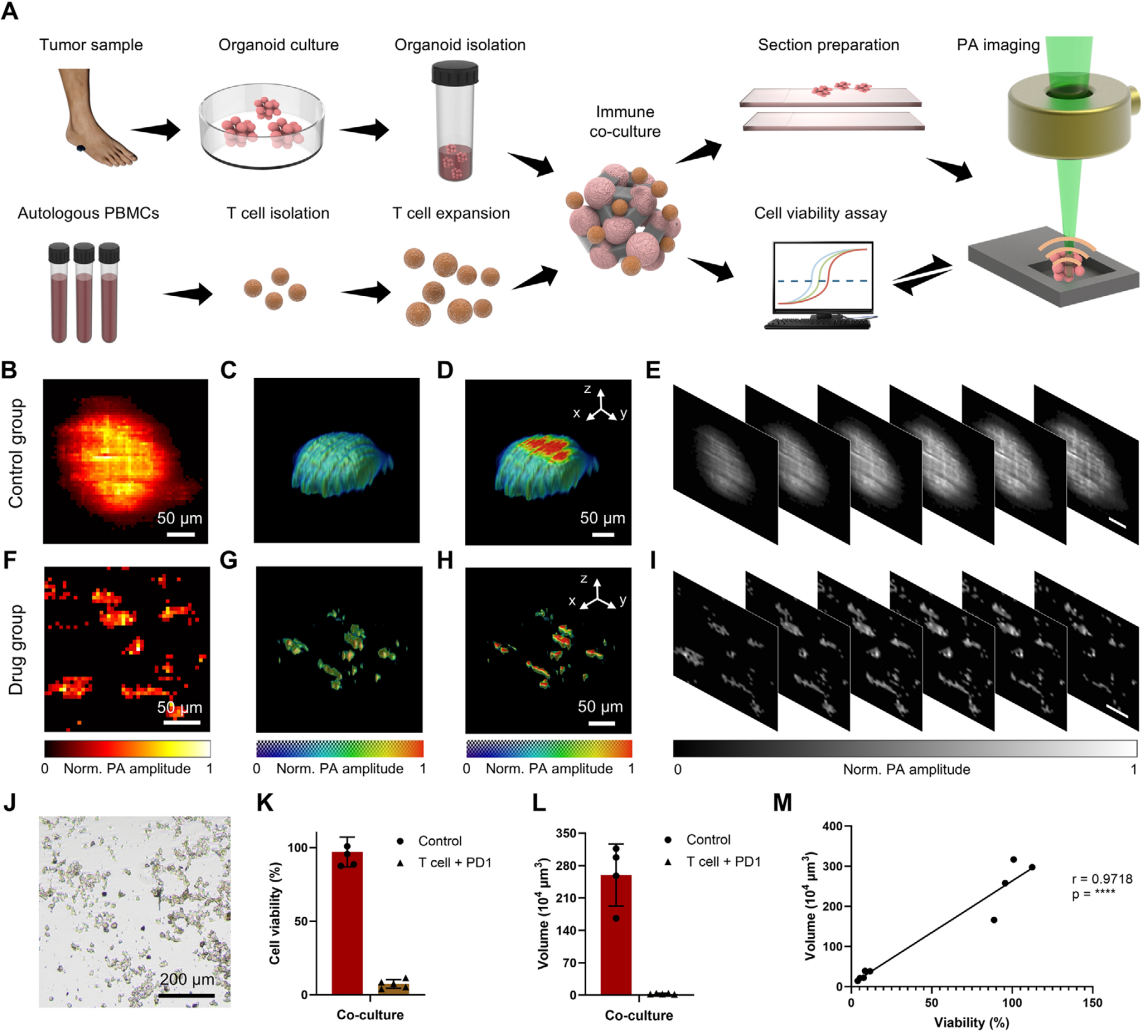
Volumetric immunotherapy drug screening using the LFOPI system. A) Workflow of 3D tumor organoids immunotherapy drug screening via LFOPI. B) Maximum z‐projections of 3D volume from control group. C) 3D visualization of the organoid from (B). D) Cross‐section of organoid shown in (C). E) Different z‐position slices through the organoid in (C). F) Maximum z‐projections of 3D volume from CD45^+^ T cell group. G) 3D visualization of the organoid from (F). H) Cross‐section of organoid shown in (G). I) Different z‐position slices through the organoid in (F). J) Bright field microscopy images of melanoma organoids co‐cultured with CD45^+^ T cells. K) Viability comparison between control and CD45^+^ T cell group. Data are presented as mean ± s.d. (*n* = 3). L) Volumetric comparison between control and CD45^+^ T cell group. Data are presented as mean ± s.d. (*n* = 3). M) Correlation between organoid volumes and viability in co‐cultured models. Scale bars in (E,I) are set at 50 µm.

Immunological drug screening constitutes a meaningful progress in the field of oncology, especially for diseases such as melanoma where traditional chemotherapy may fall short. The innovative potential of the LFOPI technique for monitoring the effectiveness of immunotherapy presents a promising avenue for understanding and improving cancer treatment strategies. With this in mind, we embarked on a study to evaluate the efficacy of our LFOPI system in visualizing and quantifying the impact of immunotherapeutic agents, specifically targeting CD45^+^ T cells in melanoma organoids. Figure 5A showcases the results from utilizing LFOPI method to perform 3D melanoma organoid CD45^+^ T cell immunotherapy drug screening. In our experimental design, patient‐derived melanoma organoids were cultured and divided into two distinct cohorts: a control group which remained untreated (Figure 5B–E), and an experimental group that was subjected to a range of immune cells (Figure 5F–I). Our LFOPI system can effectively captured the dynamic responses of these organoids to the administered treatments. Maximum z‐projections of the 3D tumor volumes for each group revealed pronounced structural differences. The organoids in the control group maintained their initial size and morphology (Figure 5B), serving as a baseline for comparison. In contrast, the organoids treated with immunotherapy exhibited significant structural changes (Figure 5F), including a drastic reduction in volume and the formation of irregular structures compared to the untreated samples. These LFOPI images illustrate areas of cell death and disintegration, highlighting the effectiveness of immunotherapy in targeting and eliminating cancer cells. The capacity of the LFOPI to capture the full depth of the organoids, with melanoma samples arrayed at various z‐depths on a glass slide for imaging (Figure 5E,I), offered an unprecedented level of detail. This approach enabled precise spatial localization of tumor regions and drug effects, ensuring that even subtle changes in organoid structure could be detected, which is essential for early intervention and comprehensive drug evaluation. Figure 5J and Figure S7 (Supporting Information) show the irregular shape of the organoids following interaction with CD45^+^ T cells, indicating the cytotoxic effects of the immune response. Following the imaging process, we conducted a quantitative analysis to correlate physical changes with biological outcomes. Cell viability assays demonstrated a clear decrease in viability among the treatment group, strongly aligning with the volumetric data obtained from LFOPI images (Figure 5K). Photoacoustic volumetric measurements complemented these findings, confirming a marked reduction in tumor volume as a result of the drug administration (Figure 5L). Of note, the traditional methods of measuring organoid volume, often based on assumptions of regular geometry and homogeneity, become less applicable after immune cell interaction, which can cause irregularity in shape and size. Our approach, using pixel‐based calculations to determine organoid volume, accounts for the irregular shapes and incomplete structures that typically result from T‐cell attacks, providing a more accurate measure of the residual organoid volumes. The overarching correlation between photoacoustic volumetric measurements and tumor cell viability confirms the utility of the LFOPI system as a formidable tool for monitoring responses to both chemotherapy and immunotherapy in 3D melanoma organoids (Figure 5M). This capability highlights the transformative potential of the LFOPI not only in assessing the immediate impact of immunotherapy drugs but also in facilitating a deeper understanding of their mechanisms. By providing a clearer 3D picture of how immunotherapy affects tumor organoids at the cellular level, our research contributes to a broader understanding of cancer biology.

## 3 Discussion

The development of 3D organoid cultures represents a major advancement for precision medicine, offering a more accurate model of in vivo tumor environments. In this study, we selected patient‐derived tumor organoids, rather than conventional melanoma spheroids, to evaluate the performance of the LFOPI system. Although multicellular spheroids derived from melanoma cell lines are widely used for basic drug screening, they lack critical features of clinical tumors. More importantly, spheroids derived from immortalized cell lines do not capture the inter‐patient variability or tumor heterogeneity that is characteristic of real‐world clinical tumors. In contrast, tumor organoids better preserve the original tumor's key characteristics and patient‐specific differences, making them a superior platform for personalized medicine. This allows the LFOPI system to demonstrate high‐resolution imaging with potential for individualized drug sensitivity evaluation.

Traditional imaging techniques, however, often fail to detect the complex volumetric changes organoids undergo during drug exposure, limiting their potential in drug screening. To address this, our study introduces a LFOPI system, a new method providing rapid, high‐resolution 3D imaging to directly observe and quantify structural changes in tumor organoids, enhancing drug efficacy assessments. The LFOPI system not only preserves the integrity and viability of organoids but also simplifies the preparation process, making it highly suitable for dynamic and repeated assessments crucial in drug development cycles. This research focused on employing LFOPI for volumetric drug screening of melanoma organoids.

While our current study utilized tumor organoids under 100 µm in diameter, optical diffraction strategies such as needle‐shaped beam illumination^[^ ^16^ ^]^ can be integrated to enhance imaging performance for larger organoids. In contrast to traditional methods that rely heavily on 2D measurements or require invasive processing, the LFOPI system enables comprehensive volumetric assessment through real‐time 3D reconstruction. By comparing LFOPI‐derived volumetric data with data from conventional imaging techniques, we observed considerable discrepancies in the quantification of drug‐induced changes. This underscores the importance of 3D reconstruction in accurately capturing drug responses. Moreover, LFOPI facilitates non‐invasive sectioning through z‐stack acquisition, revealing internal morphological alterations across different regions of an organoid, which is essential for understanding spatially heterogeneous drug responses.

The advantages of LFOPI extend beyond mere imaging. Experiments demonstrated the system capability to track dynamic changes in organoid structure following drug treatment. This was particularly evident in the comparison of treated versus untreated melanoma organoids, where treated organoids exhibited notable decreases in volume and alterations in morphology. Such changes are critical indicators of drug effectiveness, highlighting the potential of the LFOPI to serve not only as a diagnostic tool but also as a powerful instrument for real‐time monitoring of treatment responses.

The integration of LFOPI into drug screening processes also promises to enhance the reproducibility and reliability of preclinical studies. The ability to obtain consistent, repeatable 3D images ensures that drug effects are not only more accurately assessed but also better documented, facilitating comparisons across different studies and expediting the drug validation process. In conclusion, this study not only highlights the deficiencies of traditional drug screening methods in capturing the complex realities of tumor organoids but also demonstrates the profound capabilities of the LFOPI in filling these gaps. By enabling detailed, non‐invasive, and real‐time visualization of 3D tumor organoid responses to drugs, LFOPI sets a new standard in the field of cancer research. It holds the promises to advance the understanding of drug interactions within the tumor microenvironment. It also paves the way for developing more effective, personalized cancer treatments.

The current approach still has certain limitations. While the LFOPI system provides valuable insights into organoid volume and viability, it currently focuses mainly on these aspects and does not capture all the dynamic interactions and microenvironmental factors influencing organoid behavior. In future work, we plan to integrate additional imaging modalities to address these limitations. The depth penetration of the LFOPI system, although superior to many traditional optical imaging techniques, is still limited by the optical properties of the tissues. This restricts the maximum size of organoids that can be effectively imaged in their entirety, potentially excluding larger organoid models that emulate more advanced tumor stages. Therefore, overcoming tissue optical scattering and absorption to enhance penetration depth is a critical and complex challenge. Addressing this by combining multimodal imaging strategies with acousto‐optic modulation techniques can ensure accurate volumetric assessment of organoids, ultimately improving the reliability and efficacy of drug evaluation. Moreover, gaining a deeper understanding of the mechanisms underlying organoid volume changes is essential, as both drug‐induced cell death and immune‐mediated clearance can lead to reductions in volume. In our subsequent research, we plan to incorporate complementary approaches such as fluorescence imaging, immune cell infiltration markers (e.g., CD45, granzyme B, PD‐L1), and histological analysis to elucidate the specific causes of volume change. This integrated strategy will provide more precise insights into immune responses and further enhance the accuracy of volumetric drug screening in evaluating immunotherapy efficacy.

For high‐throughput drug screening, although the current imaging speed is moderate, ongoing system optimizations including the adoption of higher repetition rate laser sources and multi‐focus parallel scanning are expected to enhance both speed and resolution. Regarding the applicability of the LFOPI system to other tumor types or non‐tumor organoids, we acknowledge that melanoma organoids provide excellent photoacoustic signals at 532 nm. However, for non‐pigmented tumors or non‐tumor organoids, we recognize that the weaker photoacoustic signals, due to the absence of strong endogenous absorbers like melanin, could present challenges in terms of sensitivity and imaging quality. While these limitations exist, we believe there are potential solutions, such as using shorter wavelengths (e.g., 266 nm) to better target structures like DNA or RNA, which exhibit stronger absorption at these wavelengths.

## 4 Experimental Section

### Ethical Approvals

All experiments were approved by the Medical Ethics Committee of Xiangya Hospital, Central South University (202308636). Informed consent was obtained from all patients prior to participation in the study. For 532 nm light, the American National Standards Institute (ANSI) defines the maximum permissible exposure as 20 mJ cm^2^. The laser power is far below this limit.

### Tissue Processing

Fresh melanoma tumor tissues obtained from patients undergoing surgery for malignant melanoma were thoroughly washed to eliminate surface contaminants. The tissues were mechanically dissociated into approximately 3 mm^3^ fragments, followed by mild enzymatic digestion to produce a single‐cell suspension. The suspension was centrifuged at 1500 rpm for 5 min, and the resultant pellet was resuspended in Matrigel. After the Matrigel solidified, a specialized medium for tumor organoids was added to the culture dish. Within approximately 10 d, mature tumor organoid spheres formed within the Matrigel matrix, ready for subsequent drug treatments and co‐culture experiments.

### Tumor Organoid Culture

The resulting cell pellets were seeded into growth factor‐reduced BME gel, with each well receiving 100 µL containing no more than 1 × 105 cells. The cultures were maintained in tumor organoid medium (DMEM/F12) (Gibco) enriched with N2 (Gibco) and B27 supplements (Gibco), 10% R‐spondin‐conditioned medium (PeproTech), EGF (50 ng mL^−1^, R&D Systems), FGF‐10 (100 ng mL^−1^, R&D Systems), N‐acetylcysteine (1.25 mm, Sigma–Aldrich), Rho‐kinase inhibitor Y‐27632 (10 µm, Abmole), A83‐01 (5 µm, Sigma–Aldrich), 1× Glutamax, 10 mm nicotinamide, and 1 mm N‐acetylcysteine. Organoids were passaged using mechanical dissociation or TrypLE Express (Invitrogen), supplemented with 10 µm Y‐27632 every 1–3 weeks. The medium was refreshed every 3 or 4 d, based on the organoids’ growth dynamics. After passaging, cells were reseeded in BME and overlaid with tumor organoid medium following centrifugation with 5–10 mL of DMEM/F12 at 500 g.

### Drug Assays for Tumor Organoids

The cells were seeded in standard 96‐well cell culture plates (Corning) at a density of 1.5 × 10^4^ cells per well. Following the solidification of Matrigel, 150 µL of tumor organoid‐conditioned medium was added. Seven to ten days post‐seeding,70 µL of drug‐containing medium was introduced into each well. Three compounds along with vehicle controls were selected for drug screening, and each screen was conducted in triplicate using concentrations determined from preliminary experiments.

### Immunotherapy Testing

Matured tumor organoids were harvested from Matrigel using TrypLE Express, followed by co‐culturing with CD45^+^ T cells that had been sorted and expanded from the same patient, mixed at a ratio of 1:5 (tumor cells to lymphocytes). Co‐cultures were conducted for 48 h in the presence of T cell activation medium, utilizing 96‐well round‐bottom ultralow attachment microplates (Corning). In the experimental group, anti‐PD‐1 antibody was introduced.

### Cell Viability and Organoid Size Analysis

Cell viability was assessed using the Cell Counting Kit‐8 (CCK‐8) assay (Bimake, B34302). A tenth of the total volume of CCK‐8 reagent was added directly to the culture medium, and incubation continued for 1 to 4 h until a visible color change to orange was observed. Absorbance at 450 nm was measured using a microplate reader. Tumor organoids were visually inspected before and after drug treatment, with five representative organoids selected from each group for detailed analysis. Organoid size was assessed by measuring both the major axial length and minor axial length (considered as width), with volumes calculated using the formula: Volume = 0.5 × length × width^2^. Organoid surface area was quantified using the ImageJ software.

### Sample Preparation for Photoacoustic Imaging of Organoids and Optical Imaging

Mature tumor organoids, both intact and those showing volume changes post‐treatment or immune co‐culture, were fixed for subsequent photoacoustic imaging analysis. Tumor organoids embedded in Matrigel required digestion with TrypLE Express to release organoid clusters. Tumor organoids processed for immunotherapy testing were exempt from these steps. Organoids were then fixed in 4% paraformaldehyde (PFA) and mounted on confocal‐specific culture dishes for rapid imaging observation. During processing, we ensured consistent infiltration times and procedures across all experimental groups, resulting in uniform, 3D shrinkage of the organoids. Although shrinkage occurred, its consistency minimizes its impact on inter‐group differences. For 3D LFOPI imaging in this study, organoid specimens were placed on glass slides using a sample holder and imaged without further processing.

### Label‐Free Reflection‐Mode LFOPI

Our LFOPI system is consisted of a Q‐switched DPSS 532 nm nanosecond pulsed laser (Spot V216 Lasers, Elforlight) to illuminate the imaged object. A customized ring‐shaped ultrasonic transducer (20 MHz center frequency, 30 mm diameter, 80% −6 dB two‐way bandwidth) with a central aperture was used to detect the photoacoustic signal, which allows optical and acoustical confocal alignment. The transducer was placed 30 mm above the sample to detect the photoacoustic signals. The 532 nm laser has a pulse repetition rate of 10 kHz with a pulse duration of 2 ns. The laser beam was expanded by a pair of plano‐convex lenses and spatially filtered by a 15 µm high‐energy pinhole (P15K1, Thorlabs). The LFOPI image was acquired by scanning the water‐immersed sample mounted onto a customized 3D scanner consisting of three step motors (PA150, Zolix).

A typical scan covered an area of 200 µm × 200 µm with a step size of 5 µm, and the total acquisition time for this region was approximately 30 seconds. The detected photoacoustic signal was amplified by two low‐noise amplifiers (ZFL‐500LN+, Mini‐Circuits) and digitized by a data acquisition card (PCIE5124, National Instruments) at a 200 MHz sampling rate. The reconfigurable I/O device (PCIE‐6323, National Instruments) with a custom‐written LabVIEW (National Instruments) program was used to control and synchronize laser pulses, motor movements, and data acquisition (Figure S1, Supporting Information).

### Fabrication of the High‐Frequency Ultrasonic Transducer

The fabrication process of the high‐frequency ultrasonic transducer is outlined as follows. As shown in Figure S1 (Supporting Information), the transducer, based on lithium niobate (LNO) single crystal, has a central 6 mm diameter hole. First, lithium niobate was chosen as the piezoelectric material for acoustoelectric conversion due to its strong piezoelectric properties and low dielectric constant, making it ideal for large‐aperture sensors with electrical impedance matching (50 Ω). Second, using the material parameters, the transducer was designed with PiezoCAD, a modeling software based on the Krimboltz–Leedom–Mattaei equivalent circuit model, resulting in dimensions of 20×20 mm to meet experimental requirements. Third, a slightly larger LNO plate was ground to the appropriate thickness for its resonance frequency, and a gold (Au) electrode was sputtered onto one side using magnetron sputtering. A backing material, E‐solder 3022 (conductive silver paste, acoustic impedance ≈5.9 MRayl), was applied to the electrode side and cured before being ground down to 1 mm. The LNO and backing material stack was then cut to 20×20 mm, and a lead wire was attached to the backing material. The assembly was placed into a cylindrical aluminum housing with an SMA connector on the side, encapsulated in epoxy resin. Another Au electrode was sputtered onto the opposite side of the LNO and the aluminum casing to form a common ground. A lens ring was attached to the top, and epoxy resin was poured into it. A 34 mm diameter ball was used to create a spherical indentation, and after curing, the ball was removed. Finally, a 6 mm hole was drilled through the probe using a CNC lathe.

### Theoretical TIR

In optical‐resolution photoacoustic microscopy, it is assumed that the optical fluence is localized at a single point in space. Moreover, if the laser pulse is sufficiently short to satisfy both thermal and stress confinement conditions, we can further assume that the light fluence distribution at location **r** in time *t* behaves like a *δ* function. For each scanned position **r′**, the generated optical‐resolution photoacoustic microscopy signal *p*_0_(***r*** *,t)* can be described by the following equation:^[^ ^28^, ^29^, ^30^, ^31^, ^32^ ^]^

(1) $$p_{0}\left(r,t\right)\cdot \frac{\Gamma }{4\pi c^{2}\left|r-r^{\prime }\right|}\cdot \frac{\partial }{\partial t}\delta \left(t-\frac{\left|r-r^{\prime }\right|}{c^{2}}\right)$$

where *c* is the speed of sound, **Γ** denotes Grüneisen parameter.

The optical‐resolution photoacoustic microscopy signals *p*_temp_ (r, *t*) detected by an ultrasound transducer result from the convolution between the ultrasonic wave generated in the excited medium and the transducer's properties. In high‐speed laser scanning optical‐resolution photoacoustic microscopy, signals are detected from positions with both axial and lateral offsets from the acoustic focus. As the acoustic waves pass through the transducer's focusing element, often a plano‐concave glass lens, distortions and interferences modify the raw signals and, in turn, affect the reconstructed images.

These distortions are captured in the transducer's total impulse response TIR (**r**, *t*), which reflects the convolution of the initial pressure p_0_ (**r**, *t*) with the spatial impulse response SIR (**r**, *t*), dependent on the sensing position **r**, and the electric impulse response EIR(*t*), which is spatially invariant and reflects the effects of the electronic acquisition system:^[^ ^25^ ^]^

(2) $$p_{\mathrm{temp}}\left(r,t\right)=p_{0}\left(r,t\right)*\mathrm{SIR}\left(r,t\right)*\mathrm{EIR}\left(t\right)=p_{0}\left(r,t\right)*\mathrm{TIR}\left(r,t\right)$$

where * indicates the convolution. Finally, by determining the precise TIR (**r**, *t*) at each voxel, we can achieve:

(3) $$p_{\mathrm{recon}}\left(r,t\right)=p_{\mathrm{temp}}\left(r,t\right)*\mathrm{TI}R^{-1}\left(r,t\right)≅p_{0}\left(r,t\right)$$

where *p*_recon_ (r, *t*) is retrieved as a TIR‐corrected version of the detected pressure *p*_temp_ (r, *t*), and is rectified for signal artifacts anticipated at this position in space. In this way, *p*_recon_ (r, *t*) represents a TIR‐corrected version of the detected signal *p*_temp_ (r, *t*), compensating for signal artifacts at that specific spatial position.^[^ ^25^ ^]^

### Image Processing

To reconstruct the LFOPI images, the photoacoustic amplitude of each A‐line signal was first calculated. Through the K ‐wave toolbox in MATLAB,^[^ ^30^ ^]^ simulations were conducted on the system TIR. The axial position of the organoid surface was calculated by detecting the peak of the A‐line signal after TIR matched filter correction. The 2D maximal amplitude projection image was self‐normalized. Since the photoacoustic amplitude of the contrast is proportional to its absorption coefficient, it can be used to effectively differentiate melanin from the background. Melanin has the largest absorption coefficient at 532 nm and produces the highest photoacoustic signals.

### Statistical Analysis

The sample size of each experimental group is described in the corresponding figure caption. GraphPad Prism software was used for all statistical analyses. Quantitative data displayed as histograms are expressed as mean ± standard deviation (represented as error bars). Statistical significance was set at a *P*‐value <0.05.

### Live/Dead Staining

Live and dead cells were assessed using ViaStain AOPI Staining Solution, which contains acridine orange (AO, staining live cells in green) and propidium iodide (PI, staining dead cells in red). The live/dead ratios of tumor cells were analyzed using a Countstar MiraBF/FL Plus automatic fluorescence cell analyzer.

## Conflict of Interest

The authors declare no conflict of interest.

## Author Contributions

X.F.L., M.C., and H.S. contributed equally to this work. Conceptualization, X.F.L., M.K.C., H.S., and Ze Yu C.; methodology, X.F.L., Q.B.L., and X.Z.Y.; investigation, X.F.L., M.K.C., H.S., Q.B.L., X.Z.Y., Q.T., X.W.W., C.L.L., Zi Yan C., F.Z., and J.X.H.; writing—original draft, X.F.L., M.K.C., and H.S.; writing—review & editing, J.S., Ze Yu C., S.Z., and X.C.; funding acquisition, X.F.L., J.X., Ze Yu C., and X.C.; resources, Z.X.L., C.L.F., J.X., J.S. and S.Z.; supervision, J.S., Ze Yu C., S.Z., and X.C. All authors discussed and edited the manuscript.

## Supporting information



Supporting Information

## Data Availability

Data is available upon reasonable request.
