# Supplementary material for: Label‐Free 3D Photoacoustic Imaging of Tumor Organoids for Volumetric Drug Screening
Source: Adv Sci (Weinh). 2025 Jun 4;12(31):e17226. doi: 10.1002/advs.202417226 (PMC12376656; doi:10.1002/advs.202417226)
Supplement: Supplementary file 1 — Supporting Information [file ADVS-12-e17226-s001.pdf]

ADVANCED SCIENCE
Open Access

Supporting Information

for *Adv. Sci.*, DOI 10.1002/advs.202417226

Label-Free 3D Photoacoustic Imaging of Tumor Organoids for Volumetric Drug Screening

Xiaofei Luo, Maike Chen, Han Shan, Xize Yu, Qibo Lin, Qian Tao, Xiongwei Wei, Changling Lv, Ziyan Chen, Fan Zhuo, Xi Zhu, Jiaxing He, Zhaoxi Li, Chunlong Fei, Jing Xu, Juan Su, Zeyu Chen*, Shuang Zhao* and Xiang Chen**

ADVANCED SCIENCE
Open Access

Supporting Information

Label-Free 3D Photoacoustic Imaging of Tumor Organoids for Volumetric Drug Screening

Xiaofei Luo, Maike Chen, Han Shan, Xize Yu, Qibo Lin, Qian Tao, Xiongwei Wei, Changling Lv, Ziyan Chen, Fan Zhuo, Xi Zhu, Jiaxing He, Zhaoxi Li, Chunlong Fei, Jing Xu, Juan Su, Zeyu Chen*, Shuang Zhao* and Xiang Chen**

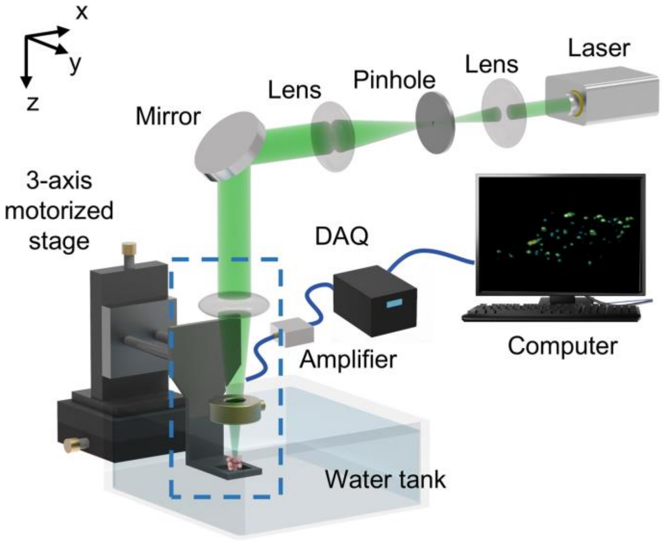

Figure S1. Schematic of the 3D scanning LFOPI system setup. The system includes the laser, lenses, pinhole, mirror, 3-axis motorized stage, DAQ, amplifier, and a computer.

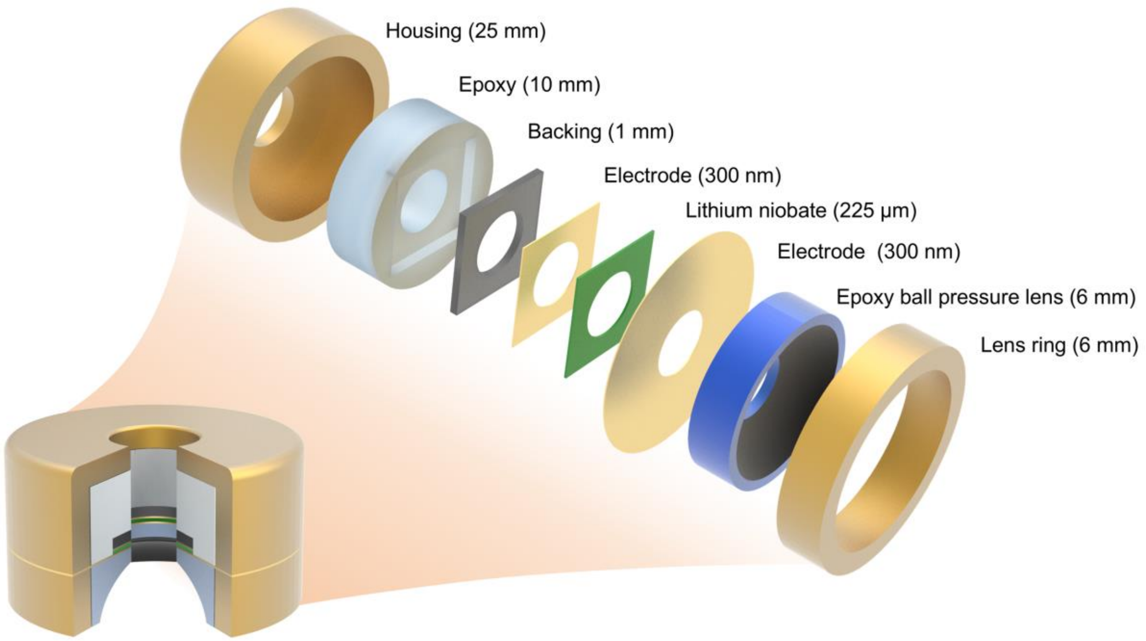

Figure S2. Exploded view of the single-element ultrasonic transducer. Schematic and exploded view of the single-element ultrasonic transducer. Thicknesses of different layers of materials are provided in brackets.

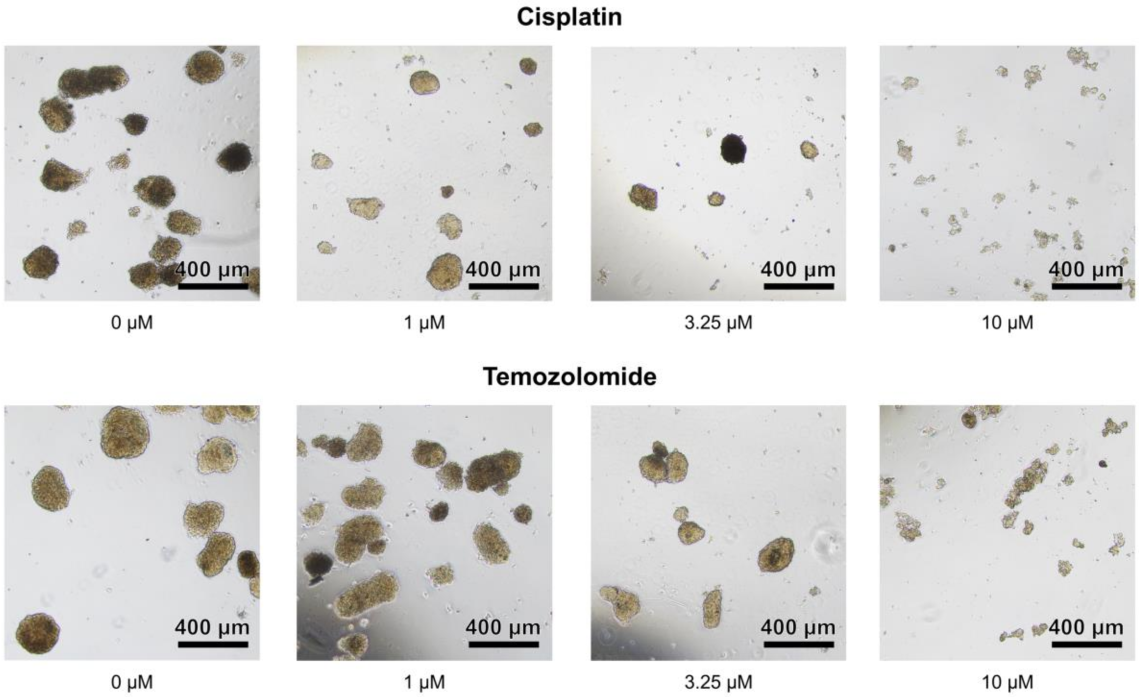

Figure S3. Bright field microscopy images of melanoma organoids treated with ascending concentrations of cisplatin and temozolomide. A Cisplatin. B Temozolomide.

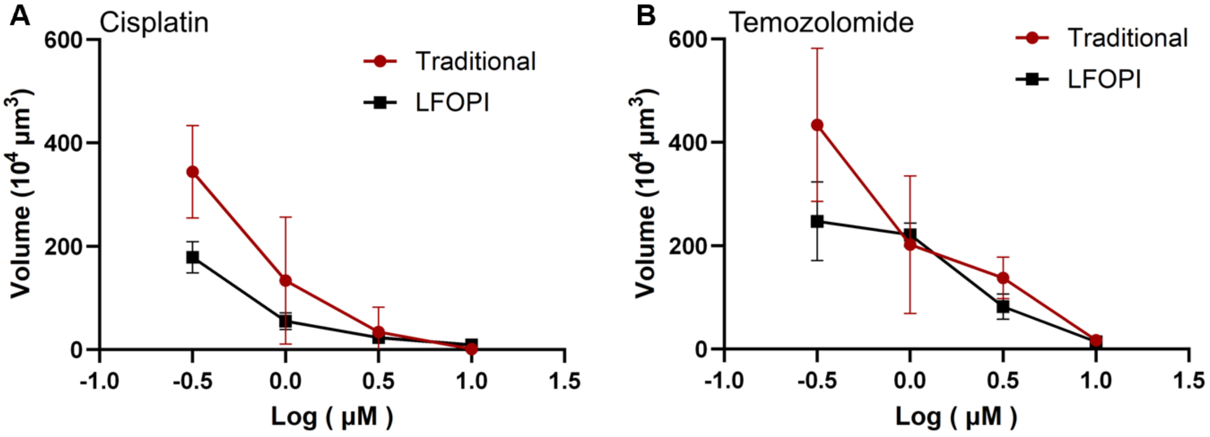

Figure S4. Organoid volume comparison between the traditional method and our LFOPI method. A Cisplatin. B Temozolomide.

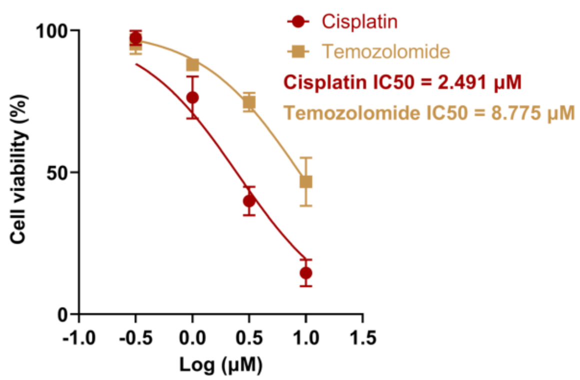

Figure S5. IC50 values of cisplatin and temozolomide. The red circle dotted line represents Cisplatin, while the yellow square dotted line represents Temozolomide.

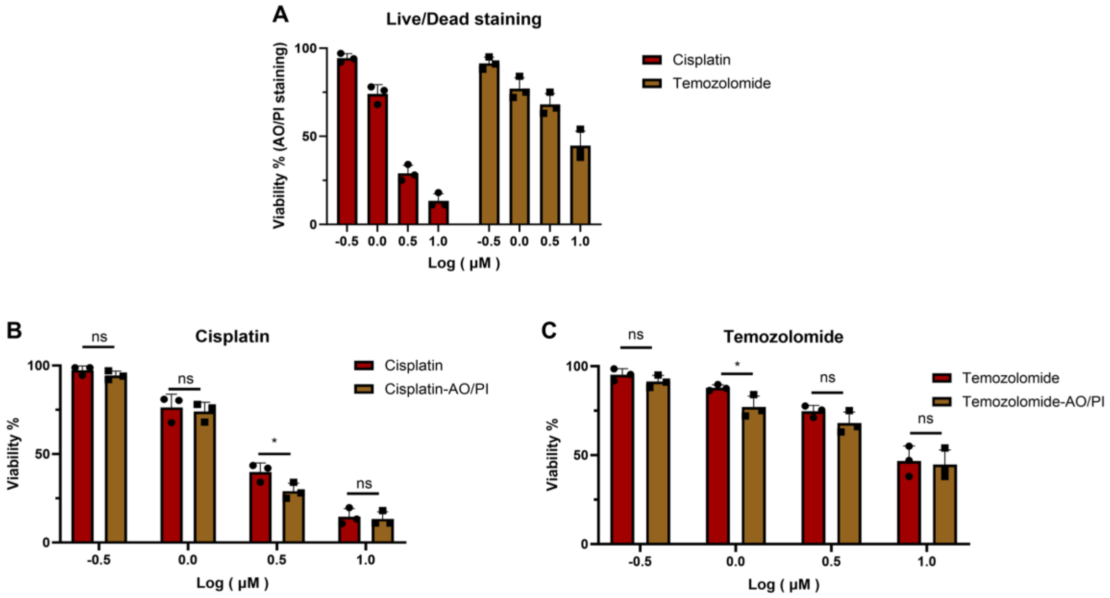

Figure S6. Comparison of Cell Viability Assays. **A)** Live/dead staining with AO/PI dye was performed on cells treated with different concentrations of Cisplatin and Temozolomide, followed by statistical analysis of the cell live rate in the treated samples. n = 3. **B)** A statistical analysis was performed on the cell viability data of the Cisplatin group obtained by AO/PI staining and CCK-8 assay. n = 3. *$p < 0.05$. (paired Student's t test); ns, no significance. **C)**. A statistical analysis was performed on the cell viability data of the Temozolomide group obtained by AO/PI staining and CCK-8 assay. n = 3. *$p < 0.05$. (paired Student's t test); ns, no significance.

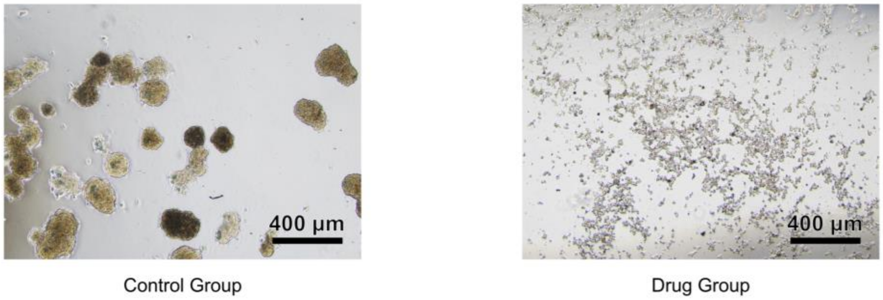

Figure S7. Bright field microscopy images of melanoma organoids co-cultured with CD45+ T cells. A Control group. B Drug group.
